# Phylogenetically Diverse *Burkholderia* Associated with Midgut Crypts of Spurge Bugs, *Dicranocephalus* spp. (Heteroptera: Stenocephalidae)

**DOI:** 10.1264/jsme2.ME16042

**Published:** 2016-06-03

**Authors:** Stefan Martin Kuechler, Yu Matsuura, Konrad Dettner, Yoshitomo Kikuchi

**Affiliations:** 1 Department of Animal Ecology II, University of BayreuthUniversitaetsstraße 30, 95440 BayreuthGermany; 2 Tropical Biosphere Research Center, University of the Ryukyus1 Senbaru, Nishihara, 903–0213Japan; 3 Bioproduction Research Institute, National Institute of Advanced Industrial Science and Technology (AIST)Hokkaido Center, 2–17–2–1 Tsukisamu-higashi, Toyohira-ku, Sapporo 062–8517Japan; 4 Graduate School of Agriculture, Hokkaido UniversityKita 9 Nishi 9, Kita-ku, Sapporo 060–8589Japan

**Keywords:** insect, bacteria, gut symbiosis, molecular phylogeny, evolution

## Abstract

Diverse phytophagous heteropteran insects, commonly known as stinkbugs, are associated with specific gut symbiotic bacteria, which have been found in midgut cryptic spaces. Recent studies have revealed that members of the stinkbug families Coreidae and Alydidae of the superfamily Coreoidea are consistently associated with a specific group of the betaproteobacterial genus *Burkholderia*, called the “stinkbug-associated beneficial and environmental (SBE)” group, and horizontally acquire specific symbionts from the environment every generation. However, the symbiotic system of another coreoid family, Stenocephalidae remains undetermined. We herein investigated four species of the stenocephalid genus *Dicranocephalus*. Examinations via fluorescence *in situ* hybridization (FISH) and transmission electron microscopy (TEM) revealed the typical arrangement and ultrastructures of midgut crypts and gut symbionts. Cloning and molecular phylogenetic analyses of bacterial genes showed that the midgut crypts of all species are colonized by *Burkholderia* strains, which were further assigned to different subgroups of the genus *Burkholderia*. In addition to the SBE-group *Burkholderia*, a number of stenocephalid symbionts belonged to a novel clade containing *B. sordidicola* and *B. udeis*, suggesting a specific symbiont clade for the Stenocephalidae. The symbiotic systems of stenocephalid bugs may provide a unique opportunity to study the ongoing evolution of symbiont associations in the stinkbug-*Burkholderia* interaction.

Most members of the taxon Heteroptera, which includes 42,300 described species (12), display mutualistic relationships with diverse symbiotic bacteria (6, 30). While some stinkbug species of the families Lygaeidae, Artheneidae, Blissidae, and Cimicidae harbor their endosymbionts intracellularly in specific organs, called bacteriomes or mycetomes (16, 36–38, 43, 57), most phytophagus stinkbugs, particularly members of the infraorder Pentatomomorpha, extracellularly accommodate their symbiotic bacteria either in the lumen of the swollen part of the midgut (26, 60) or in the lumen of the separated sac-like tissues of the posterior midgut, called crypts or ceca (6, 54, 68).

In plant-sucking stinkbugs of the superfamily Pentatomoidea (Heteroptera: Pentatomomorpha), species of the families Acanthosomatidae, Cydnidae, Parastrachiidae, Pentatomidae, Plataspidae, Scutelleridae, and Urostylididae harbor specific bacterial symbionts, which belong to distinct lineages in *Gammaproteobacteria*, indicating multiple evolutionary origins of symbiotic associations (2, 3, 13, 15, 17, 19, 23–25, 31, 34, 35, 44, 51–53). The gut symbionts of the Pentatomoidea are typically transmitted vertically by specific, postnatal transmission mechanisms, such as the bacterial contamination of the egg surface during egg deposition (1, 31, 53, 55), the excretion of a bacteria-containing mucus or jelly onto the egg mass (18, 25), and the deposition of bacteria-containing capsules together with the eggs (9, 14, 15, 47).

In contrast, most representatives of the superfamilies Lygaeoidea and Coreoidea are associated with the betaproteobacterial symbionts of a specific clade in the genus *Burkholderia*, called the “stinkbug-associated beneficial and environmental (SBE)” group (4, 10, 22, 28, 29, 32, 33, 50). Based on a series of comprehensive studies on the coreoid species, *Riptortus pedestris* (Coreoidea: Alydidae), it has been reported that *Burkholderia* symbionts are not transmitted vertically, but are acquired anew by nymphal insects from the environment every generation (29), whereas partial vertical transmission of the *Burkholderia* symbiont has been reported in chinch bugs (4, 22). Due to the transmission mechanism, the phylogeny of the *Burkholderia* symbiont does not mirror the phylogeny of the host insects, but symbionts form a coherent group with soil-isolated strains in an intermixed manner (28, 29, 32), indicating an alternating host–symbiont relationship. In addition, a recent study revealed that representatives of the family Largidae of the superfamily Pyrrhocoroidea, a monophyletic sister taxon of Coreoidea and Lygaeoidea (67), are also associated with *Burkholderia* symbionts (61). However, these largid species are, in contrast to lygaeoid/coreoid species, consistently associated with *Burkholderia* strains of the so-called “plant-associated beneficial and environmental (PBE)” group, which are phylogenetically distinct from the SBE strains (63). In the PBE-group, stinkbug-associated strains do not form a monophyletic cluster, but are intermixed with soil-isolated and plant-associated strains, also indicating a promiscuous host–symbiont association in pyrrhocorid stinkbugs.

The family Stenocephalidae of the superfamily Coreoidea is a small stinkbug group, all members of which live and feed on various species of the Euphorbiaceae, commonly known as spurges (58). Landsbury (39, 40) identified two genera (*Dicranocephalus* and *Psotilnus*) and 36 species, whereas Moulet (45, 46) considered only one genus (*Dicranocephalus*) and 16 valid species. Although the group is widely distributed, most are known from the tropics and subtropics of the Eastern Hemisphere, including Australia. This small family is of special taxonomic interest because it shows characteristics that are transitional between Coreoidea (*e.g.*, numerous hemelytral veins and a four-lobed salivary gland) and Lygaeoidea (*e.g.*, laciniate ovipositor and an XY chromosome) (11, 12, 58). Therefore, the phylogenetic position of Stenocephalidae was controversial for a long time (11). In addition to the taxonomical importance of this family, its gut symbiotic association has not yet been characterized.

The objective of this study was to analyze and compare the bacterial populations of midgut crypts in the stenocephalid species *Dicranocephalus albipes*, *D. agilis*, and *D. medius* (Fig. 1A) from Europe, and *D. lateralis* from Japan (Heteroptera: Stenocephalidae). The phylogenetic position of the bacterial gut symbiont was elucidated by analyses of 16S rRNA and *gyrB* gene sequences. The localization as well as morphological characteristics of the gut symbiont of *D. medius* was investigated in detail by fluorescence *in situ* hybridization (FISH) and transmission electron microscopy (TEM), respectively. The results obtained revealed that a novel clade of *Burkholderia* is associated with the stenocephalid species.

## Materials and Methods

### Insects

Adults and nymphs of *D. agilis*, *D. albipes*, *D. lateralis*, and *D. medius* were collected from their host plants (*Euphorbia* spp.) in Europe and Japan (Table 1). The European bug species were brought alive to the laboratory and dissected in phosphate-buffered saline (PBS: 137 mM NaCl, 2.7 mM KCl, 8.1 mM Na_2_HPO_4_, 1.5 mM KH_2_PO_4_ [pH 7.4]), while samples of *D. lateralis* preserved in acetone were dissected in the same manner. The isolated midgut tissues were subjected to DNA extraction, cloning, and sequencing. Furthermore, several individuals of *D. medius* were used in symbiont visualization via FISH and an ultrastructure analysis by TEM.

### Histology

In order to prepare tissue sections, the dissected midgut tissues of *D. medius* were fixed in 4% paraformaldehyde overnight, followed by a washing step in 0.5×PBS and 48% ethanol (v/v), serial dehydration in ethanol (70%, 90%, 2×100%), and a final embedding step in resin Unicryl™ (Plano GmbH, Germany). Serial sections (2 μm) were cut using a Leica Jung RM2035 rotary microtome (Leica Instruments GmbH, Wetzlar, Germany), mounted on epoxy-coated glass slides, and subjected to FISH.

### FISH

Several eubacterial probes and a symbiont-specific probe (Table S1) were used to detect gut symbionts in *D. medius* midgut tissue. The specific probe was designed on a symbiont specific position in the 16S rRNA gene alignment and verified on probeCheck (42). In addition, a nonsense probe complementary to the bacterial probe EUB338 was used as a negative control of the experiment (Table S1). Tissue sections were incubated with a hybridization buffer (20 mM Tris-HCl [pH 8.0], 0.9 M NaCl, 0.01% sodium dodecyl sulphate [SDS], 20% formamide) containing 10 pmole mL^−1^ each of the fluorescent probes, kept at 46°C for 90 min, rinsed with a washing buffer (20 mM Tris-HCl [pH 8.0], 450 mM NaCl, 0.01% SDS), mounted with an anti-photobleaching solution (VectaShield Mounting Medium; Vector Laboratories, Peterborough, UK), and viewed under a fluorescent microscope (Axioplan 2 imaging, Zeiss).

### TEM

The dissected tissues of *D. medius* were fixed in 2.5% glutaraldehyde in 0.1 M cacodylate buffer (pH 7.3) for 1 h, embedded in 2% agarose gel, and fixed again in 2.5% glutaraldehyde in 0.1 M cacodylate buffer (pH 7.3) overnight. The tissue was washed in 0.1 M cacodylate buffer for 20 min three times. Following postfixation in 2% osmium tetroxide for 2 h, the sample was washed and stained *en bloc* in 2% uranyl acetate for 90 min. After fixation, the tissue was dehydrated serially in ethanol (30%, 50%, 70%, 95%, and 3×100%), transferred to propylene oxide, and embedded in Epon. Ultrathin sections (70 nm) were cut using a diamond knife (Micro-Star, Huntsville, TX) on a Leica Ultracut UCT microtome (Leica Microsystems, Vienna, Austria). Ultrathin sections were mounted on pioloform-coated copper grids and stained with saturated uranyl acetate, followed by lead citrate. The sections were viewed using a Zeiss CEM 902 A transmission electron microscope (Carl Zeiss, Oberkochen, Germany) at 80 kV.

### DNA extraction, cloning, and sequencing

The DNA of the dissected midgut crypts was extracted using the High Pure PCR Template Preparation Kit (Roche) following the manufacturer’s instructions. A 1.5-kb segment of the bacterial 16S rRNA gene was PCR amplified using the universal primers and a 0.65-kb segment of the bacterial *gyrB* (gyrase subunit B) gene was amplified for additional symbiont characterization (Table S1). In host phylogenetic analyses, several mitochondrial gene fragments were amplified: a 1.5-kb segment of cytochrome c oxidase subunit I (*COI*/*coxI*), a 0.93-kb segment of ubiquinone oxidoreductase subunit 1 (*ND1*/*nad1*), a 1.14-kb segment of cytochrome b (*cytB*/*cob*), and a 0.29-kb segment of ubiquinone oxidoreductase subunit 6 (*ND6*/nad6), respectively (Table S1).

All PCR reactions were performed on a Biometra thermal cycler with the following program: an initial denaturation step at 94°C for 3 min, followed by 34 cycles at 94°C for 30 s, 50°C for 2 min, and 72°C for 1 min. A final extension step at 72°C for 10 min was included. All PCR products of bacterial gene amplification were cloned using the CloneJET™ PCR Cloning Kit (Thermo Fisher). Cloned inserts offering PCR products of the correct length were examined by restriction fragment length polymorphism (RFLP). Inserts were digested by the restriction endonucleases *Rsa*I and *Hha*I. Eighty clone sequences for the 16S rRNA gene and 91 clone sequences for the *gyrB* gene were chosen for Sanger sequencing, respectively (Table 2 and S2). Amplified mitochondrial gene segments (*COI*, *ND1*, *cytB*, and *ND6*) of the host were subjected to direct Sanger sequencing with suitable PCR primers.

### Phylogenetic analyses

Clone sequences of gut bacteria were classified into operational taxonomic units (OTUs) using macqiime v1.9.1 (7) based on the furthest-neighbor algorithm with a >99% identity threshold for 16S rRNA gene sequences and >98% for *gyrB* gene sequences (Table 2 and S2). According to the OTUs calculation, high-quality sequences of the 16S rRNA and *gyrB* genes were aligned using the ClustalW algorithm in MEGA 6 (64) and edited manually. In the phylogenetic analysis of host insects, the mitochondrial *COI*, *ND6*, *cytB*, and *ND1* gene sequences of allied heteropteran insects were retrieved from GenBank and aligned with the sequences of *Dicranocephalus* spp. by MAFFT v7.212 (G-INS-i) (27). After manually editing gap-containing sites, *COI*, *ND6*, *cytB*, and *ND1* gene sequences were concatenated and used in subsequent analyses.

Phylogenetic trees were reconstructed under the Tamura 3-parameter (16S rRNA gene) and GTR+I+G model (*gyrB* and *COI*+*ND6*+*cytB*+*ND1*) of nucleotide substitution by the maximum likelihood (ML) method using MEGA 6. Additionally, the neighbor-joining (NJ) method was executed for phylogenetic analyses in MEGA 6. The bootstrap values of 1,000 replicates for all internal branches were calculated. A likelihood ratio test was also performed using MrModeltest V.2.3 (48) to find the best-fitting models for the Bayesian analysis. The Akaike criterion selected the GTR+I+G model for bacterial 16S rRNA, *gyrB*, and host *COI*+*ND6*+*cytB*+*ND1* gene data. Under the evolutionary model, a Bayesian analysis with MrBayes (v.3.2.6) (21) was performed with four simultaneous Markov chains for each dataset. Regarding 16S rRNA, *gyrB*, and *COI*+*ND6*+*cytB*+*ND1* gene data, 1,000,000 generations were used; 1,000 trees were obtained (samplefreq=1,000) and the first 250 of these were considered to be the ‘burn in’ and discarded.

### Nucleotide sequence accession numbers

The DNA sequences of bacterial 16S rRNA and the *gyrB* genes and host *COI*, *ND6*, *cytB*, and *ND1* gene sequences determined in this study were deposited in the DDBJ/EMBL/GenBank nucleotide sequence databases under the accession numbers (LT221673–LT221855), respectively.

## Results

### *Dicranocephalus* species develop midgut crypts filled with rod-shaped bacteria

**Fig. 1:**
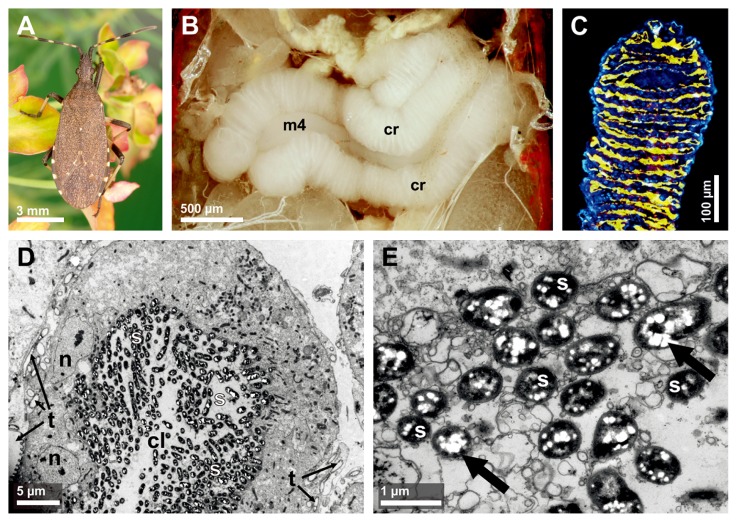
A representative of the family Stenocephalidae associated with *Burkholderia* symbionts in their midgut crypts. (A) An adult female of *Dicranocephalus medius* sitting on its *Euphorbia* host plant. (B) Dissected midgut of the fourth section (m4) with two rows of crypts (cr). (C) Fluorescence *in situ* hybridization of symbionts in midgut crypts stained with the specific *Burkholderia* probe (yellow) and DAPI (blue). (D) Transmission electron microscopy of thin sections through the midgut crypts, including the betaproteobacterial *Burkholderia* symbionts of *D. medius*. The crypt lumen (cl) is completely filled with symbionts (s), surrounded by a monolayer of nucleated (n) insect cells and numerous trachea (t). (E) High magnification of the *Burkholderia* symbionts (s). White storage granules (black arrows) are presumably cellular reserve materials.

Histological analyses revealed that the stenocephalid species *D. agilis*, *D. albipes*, *D. medius*, and *D. lateralis* possess a voluminous, white-colored midgut region with numerous crypts in two rows at the fourth midgut section (Fig. 1B). Typically, females had larger crypts than the smaller males. Field-collected *D. medius* nymphs of the 2^nd^ instar and later instars also offered well-developed midgut crypts, while no nymph of the 1^st^ instar was found in field collections in this study. When cross-sections of the midgut crypts were subjected to FISH with fluorescently labeled oligonucleotide probes specific to the gut symbiont, intense signals from symbionts were detected primarily in the lumen of the midgut crypts (Fig. 1C). Signals of a weak fluorescence were also observed from the lumen of the midgut main tract (data not shown). Ultrastructural examinations of the midgut crypts revealed that the lumen of the crypts was filled with rod-shaped bacteria (Fig. 1D). No intracellular bacteria were detected. Many of these rod-shaped bacteria contained multiple vesicles of weak electron density (Fig. 1E), which were presumably storage granules. A number of tracheal cells were observed between the single crypts (Fig. 1D), indicating a high rate of gas exchange and metabolic activity in the symbiotic tissue.

### Diverse *Burkholderia* strains are associated with *Dicranocephalus* species

Fourteen DNA samples of midgut crypts from the four species were subjected to PCR amplification, cloning, and Sanger sequencing of a 1.5-kb 16S rRNA gene fragment and 0.65-kb *gyrB* gene fragment (Table 1). The top BLAST hits of our DNA datasets (80 of the 16S rRNA gene, and 91 of the *gyrB* clone sequences) revealed a high concordance with *Burkholderia* species and the clones were classified into 10 OTUs for the 16S rRNA gene (sequences showing >99% identity were designated as a single OTU) and 11 OTUs for *gyrB* (sequences showing >98% sequence identity were designated as a single OTU) (Table 2 and S2).

The genus *Burkholderia*, which currently includes more than 90 species (http://www.bacterio.net/burkholderia.html, accessed September 23, 2015), has been divided into at least three phylogenetically and ecologically distinct clades. The first clade consists of human, animal, and plant pathogens and is designated as the BCC&P (“*B. cepacia* complex and *B. pseudomallei*”) group (8, 56, 59); the second clade includes plant growth-promoting rhizobacteria and nodule-forming plant symbionts, assigned as the PBE (“plant-associated beneficial and environmental”) group (59), which also contains a recently described subclade, called the iPBE (“insect- and plant-associated beneficial and environmental”) group (63); and the third clade is described as the SBE (“stinkbug-associated beneficial and environmental”) group (22, 35), containing free-living soil *Burkholderia* strains, leaf-gall symbionts of *Psychotria* plants, and a number of gut symbionts of Coreoidea and Lygaeoidea stinkbugs.

**Fig. 2:**
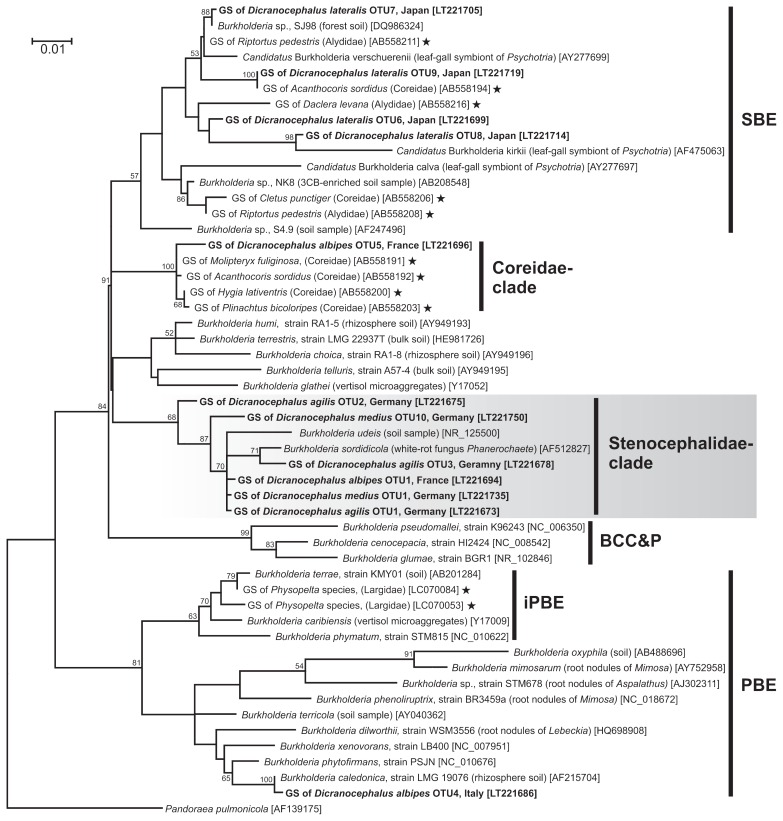
Molecular phylogeny of the gut symbiotic bacteria of stenocephalid species based on 16S rRNA gene sequences. The tree displays a maximum likelihood (ML) phylogeny of ten OTUs (>99% sequence identity) of the gut symbiotic bacteria (GS) identified from *Dicranocephalus albipes*, *D. agilis*, *D. lateralis*, and *D. medius* together with selected representatives of the different *Burkholderia* groups. Depending on the species and their collecting site, the gut symbionts of Stenocephalidae clustered in different *Burkholderia* subgroups. The alignment of 1,394 nucleotide sites of the bacterial 16S rRNA gene was used. The gut symbionts of the stenocephalid species are shown in bold. The origins or sources of the isolation of *Burkholderia* strains/sequences are represented in parentheses. Accession numbers in the DNA database (DDBJ/EMBL/GenBank) are shown in square brackets. *Burkholderia* gut symbionts of other stinkbug families are marked with an asterisk. The major *Burkholderia* clades (BCC&P, SBE, and PBE) including the subclade “insect-associated PBE (iPBE)” and “Stenocephalidae-clade” described here are indicated on the right. Bootstrap values higher than 50% are depicted at the nodes. A phylogeny of *gyrB* gene sequences from stenocephalid symbionts and other *Burkholderia* strains/sequences is shown in Fig. S1. Bayesian (MrBayes) and neighbor-joining (NJ) analyses gave essentially the same results (data not shown).

Phylogenetic analyses based on 16S rRNA gene sequences showed that the *Burkholderia* OTUs detected from the midgut crypts of the stenocephalid stinkbugs were placed in three distinct groups: PBE, SBE, and another distinct cluster that contains *B. sordidicola* and *B. udeis* with a 68% support value (the ML tree is shown in Fig. 2; NJ and Bayesian trees showed basically identical topologies). The last novel cluster is designed here as the “Stenocephalidae-clade”. Depending on the species and source of collection, stenocephalid bugs were associated with different *Burkholderia* strains (Fig. 2, S1 and Table 2, S2). For example, *Burkholderia* from one individual of *D. albipes* collected in Italy were placed close to rhizobacteria and nodule symbionts in the PBE-group, whereas *Burkholderia* from another *D. albipes* individual collected in France clustered together with *Burkholderia* from *D. medius* and *D. agilis* in the “Stenocephalidae-clade”. In addition, all *Burkholderia* from the Japanese species *D. lateralis* clustered in the SBE-group, which did not correspond to any *Burkholderia* OTUs from European stenocephalid bugs. The phylogenetic tree based on *gyrB* sequences showed basically the same clusters as those estimated by 16S rRNA sequences (Fig. S1). Notably, the “Stenocephalidae-clade” was also recognized in the *gyrB* tree with a high support value of 100%.

### Phylogenetic placement of the family Stenocephalidae in the infraorder Pentatomomorpha

**Fig. 3:**
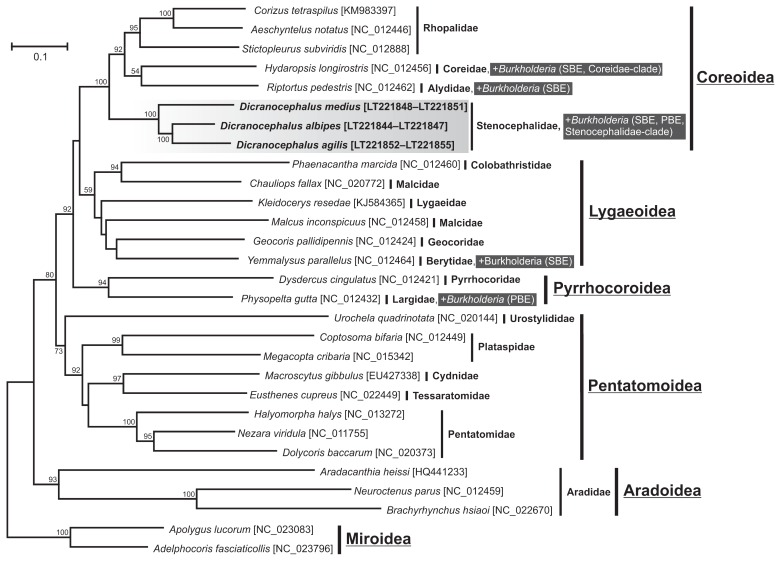
Phylogenetic position of the family Stenocephalidae within the infraorder Pentatomomorpha and their associated *Burkholderia* symbionts. The ML tree illustrates the position of Stenocephalidae as a basal group within the superfamily Coreoidea. In total, 3,996 sites of concatenated mitochondrial *COI* (1,533 bp), *ND6* (417 bp), *cytB* (1,125 bp), and *ND1* (921 bp) protein coding gene sequences of 29 species were used. The mirioid species *A. lucorum* and *L. lineolaris* were used as outgroups. Accession numbers in the DNA database (DDBJ/EMBL/GenBank) are shown in square brackets. Bootstrap values higher than 50% are depicted at the nodes. Bayesian (MrBayes) and neighbor-joining (NJ) analyses gave essentially the same results (data not shown).

The ML tree of the members of the stinkbug infraorder Pentatomomorpha revealed that the family Stenocephalidae, represented by *Dicranocephalus* species used in this study, were placed into the superfamily Coreoidea (Fig. 3). Similarly, when the concatenated alignment of *COI*+*ND6*+*cytB*+*ND1* or separate mitochondrial gene alignments were used for the analysis, all resulted in the same placement of the Stenocephalidae into the superfamily Coreoidea.

## Discussion

This study demonstrated that the stenocephalid species *D. agilis*, *D. albipes*, *D. medius*, and *D. lateralis* possess a voluminous, white-colored midgut region with numerous crypts in two rows at the fourth midgut section (Fig. 1), as reported in the coreoid families Coreidae and Alydidae (32). It has repeatedly been reported in diverse stinkbug species that the elimination of symbiotic bacteria results in retarded growth, reduced body size and fecundity, high mortality, and/or abnormal body coloration of the host insects (1, 3, 4, 9, 15, 19, 25, 31, 35, 53, 62, 65), indicating that symbiotic bacteria play a pivotal metabolic role in stinkbug hosts. Although the biological function of the symbionts in stenocephalid bugs remains unclear, the well-developed symbiotic organ (Fig. 1B) and high symbiont density in the midgut crypts (Fig. 1C) strongly suggest a positive influence on host development and reproduction.

Members of the superfamilies Coreoidea and Lygaeoidea are commonly associated with the SBE-group *Burkholderia* in the midgut crypts (4, 22, 32, 50). In the superfamily Coreoidea, members of the family Coreidae are also associated with a specific group of *Burkholderia*, called the “Coreidae-clade,” which is closely related to the SBE-group (32) (also see Fig. 2). Although our results for the Japanese species *D. lateralis* are consistent with these findings, symbionts of European stenocephalid stinkbugs reveal a loosening of this pattern. In addition to the SBE-group and Coreidae-clade *Burkholderia*, we demonstrated that European stenocephalid stinkbugs are consistently associated with a novel clade of the genus *Burkholderia* (Table 2 and S2); this clade consists of the insect symbionts and environmental species, *B. sordidicola* and *B. udeis* (41, 66), and are here designated as “Stenocephalidae-clade” *Burkholderia* (Fig. 2). These Stenocephalidae-clade *Burkholderia* may be highly specialized in the stenocephalid species and play a major role in the hosts. Although PBE-group *Burkholderia* was only detected once from *D. albipes*, it emphasizes the possibility that PBE-group *Burkholderia* are also a pivotal entity in the gut symbiosis of stenocephalid bugs, as demonstrated in the lygaeoid family Blissidae (4, 22) and the family Largidae of the superfamily Pyrrhocoroidea (63).

Recent studies have demonstrated that the *Burkholderia* symbionts of the bean bug *Riptortus pedestris* (Coreoidea: Alydidae) and allied-coreoid and -lygaeoid species were orally acquired by nymphal insects from the environment every generation (4, 10, 22, 29, 33), most likely from the rhizosphere of their food plants. This is a flexible system that allows for the horizontal acquisition of environmental bacteria, in contrast to the specific vertical transmission mechanism (*e.g.*, egg smearing and capsule transmission) described in most representatives of the stinkbug superfamily Pentatomoidea (14, 31, 53) and other insects (*e.g.*, reviewed in 5). Our results lead to the hypothesis that stenocephalid bugs also horizontally acquire *Burkholderia* symbionts from ambient environments every generation based on the following evidence: (1) the symbionts did not form a monophyletic group, but phylogenetically diverse *Burkholderia* were associated with the species (Fig. 2 and S1); (2) multiple infections of different *Burkholderia* strains was frequently detected (Table 2 and S2); (3) the symbionts formed a cluster with culturable, free-living *Burkholderia* species/strains (Fig. 2 and S1).

The phylogenetic placement of the family Stenocephalidae is a long-standing question in the taxonomy of the stinkbug infraorder Pentatomomorpha (11). Our phylogenetic analysis of stinkbugs clearly demonstrated that the family Stenocephalidae is a part of the superfamily Coreoidea with robust support values (Fig. 3). The phylogenetic tree is basically agreeable with a more extensive analysis based on complete mitochondrial genomes (67), with minor exceptions in the placement of the superfamily Pyrrhocoroidea and the different clustering of two Malcidae species within the superfamily Lygaeoidea. Nevertheless, given this phylogenetic position of the Stenocephalidae, it is assumed that the stenocephalid species are not only associated with SBE-group *Burkholderia*, similar to other members of the superfamily Coreoidea, but have also established a symbiotic relationship with “Stenocephalidae-clade” *Burkholderia* during the evolution of the insect lineage.

The discrepancy in *Burkholderia* symbionts harbored in different *Dicranocephalus* species, *e.g.*, the specific associations between Japanese *D. lateralis* and SBE-group *Burkholderia* and between European *Dicranocephalus* species and “Stenocephalidae-clade” *Burkholderia*, may be explained by ectogenous and/or endogenous factors. If the environmental soil inhabited by *Dicranocephalus* bugs is dominated by only one specific group of *Burkholderia*, the host-symbiont association pattern may be in a region-dependent manner. Alternatively, all *Burkholderia* groups are ubiquitous, but the insects may select the symbiont species or strains internally. A recent study in the bean bug *R. pedestris* discovered an intestinal-specific organ, called the “constricted region”, for symbiont sorting (49). This organ was also found in the *Dicranocephalus* species (data not shown), strongly suggesting bacterial gut sorting in this species, which may play a role in the establishment of *Burkholderia* specificity. These ectogenous and endogenous hypotheses may be tested using the reciprocal exchange of symbionts between the Japanese and European *Dicranocephalus* species.

Besides the fundamental questions about the transfer and establishment of stenocephalid-*Burkholderia* symbionts, further analyses in respect to different collection sites of stenocephalid bugs and several additional Coreoidea/Lygaeoidea species from Europe and other continents in the world are necessary in order to elucidate which *Burkholderia* strains, in principle, have the ability to establish a stable symbiotic relationship with stinkbugs. Notably, it was recently demonstrated that the ongoing evolution of obligate symbiotic gut bacteria from environmental free-living bacteria occurred in natural pentatomid stinkbug populations (20). A worldwide screening of stenocephalid species may provide a unique opportunity to study the currently ongoing evolution of expanding symbiont associations in stinkbug-*Burkholderia* symbioses.

## Supplementary Material



## Figures and Tables

**Table 1:** Samples of stenocephalid bugs used for cloning and/or sequencing in this study.

Species	Insect ID	Instar	Sex^a^	Collection location	Collection date	Collector	Accession No.	
*Dicranocephalus agilis*							*16S rRNA*	*gyrB*
	Dag1	4th	–	Lindow (Mark), Germany	Jun 13, 2014	C. Morkel	LT221673–LT221676	LT221834–LT221835
	Dag2	4th	–	“	Jun 13, 2014	“	LT221677–LT221679	LT221836–LT221843
*Dicranocephalus medius*								
	Dme1	Adult	M	Bayreuth, Germany	Apr 21, 2011	S. Kuechler	LT221738	LT221800
	Dme2	Adult	M	“	May 30, 2013	“	LT221739–LT221741	LT221817–LT221819
	Dme3	Adult	F	“	May 30, 2013	“	LT221742–LT221743	LT221820–LT221821
	Dme4	Adult	M	“	Jun 04, 2014	“	LT221744–LT221752	LT221822–LT221833
	Dme5	Adult	F	“	Jun 04, 2014	“	LT221724–LT221730	LT221753–LT221760
	Dme6	Adult	F	“	Jun 16, 2014	“	LT221731–LT221737	LT221761–LT221767
*Dicranocephalus lateralis*								
	Dla1	Adult	F	Ishigaki Is., Japan	Jul 28, 2002	K. Kohno	LT221697–LT221702	LT221768–LT221778
	Dla2	Adult	M	“	Jul 28, 2002	“	LT221703–LT221712	LT221779–LT221781
	Dla3	Adult	M	“	Jul 28, 2002	“	LT221713–LT221714	LT221782–LT221789
	Dla4	–	–	“	Sep 10, 2009	T. Hosokawa	LT221715–LT221723	LT221790–LT221799
*Dicranocephalus albipes*								
	Dal1	Adult	M	Talamone, Italy	Jul 27, 2010	S. Kuechler	LT221680–LT221693	LT221801–LT221813
	Dal2	Adult	M	La Garde-Freinet, France	Sep 15, 2011	S. Kehl	LT221694–LT221696	LT221814–LT221816

a F, female; M, male; –, undetermined

**Table 2:** The number of 16S rRNA gene clones in each OTU subgroup of *Burkholderia* symbionts.

Species	Insect ID	Stenocephalidae-clade			Coreidae-clade			SBE clade			PBE clade	Total
			
		OTU1	OTU2	OTU3	OTU4	OTU5	OTU6	OTU7	OTU8	OTU9	OTU10	
*Dicranocephalus agilis*												
	Dag1	3	1	–	–	–	–	–	–	–	–	4
	Dag2	–	–	3	–	–	–	–	–	–	–	3
*Dicranocephalus medius*												
	Dme1	1	–	–	–	–	–	–	–	–	–	1
	Dme2	7	–	–	–	–	–	–	–	–	–	7
	Dme3	7	–	–	–	–	–	–	–	–	–	7
	Dme4	3	–	–	–	–	–	–	–	–	–	3
	Dme5	2	–	–	–	–	–	–	–	–	–	2
	Dme6	8	–	–	1	–	–	–	–	–	–	9
*Dicranocephalus lateralis*												
	Dla1	–	–	–	–	–	4	2	–	–	–	6
	Dla2	–	–	–	–	–	–	10	–	–	–	10
	Dla3	–	–	–	–	–	–	1	1	–	–	2
	Dla4	–	–	–	–	–	–	–	–	9	–	9
*Dicranocephalus albipes*												
	Dal1	–	–	–	–	–	–	–	–	–	14	14
	Dal2	2	–	–	–	1	–	–	–	–	–	3
	OTU total	33	1	3	1	1	4	13	1	9	14	80
