# Supplementary material for: Phylogenetically Diverse Burkholderia Associated with Midgut Crypts of Spurge Bugs, Dicranocephalus spp. (Heteroptera: Stenocephalidae)
Source: Microbes Environ. 2016 Jun 3;31(2):145–53. doi: 10.1264/jsme2.ME16042 (PMC4912149; doi:10.1264/jsme2.ME16042)
Supplement: Supplementary file 1 [file 31_145_s1.pdf]

Table S1. PCR primers and probes used in this study.

Target organism	Target gene	Primer name	Primer sequence (5'-3')	Product size	Reference[1]
Primers for amplifying, cloning and sequencing					
Bacteria	16S rRNA	07F<br>1507R	AGAGTTTGATCMTGGCTCAG<br>TACCTTGTTACGACTTCAC	1.5 kb	[1]
Burkholderia strains	*gyrB*	*gyrB*135F<br>*gyrB*850R	ACCGGTCTGCAYCACCTCGT<br>TCGTTGTAGCTGTCGTTCCACTGC	0.65 kb	modified from [2]
Insect (Stenocephalidae)	*cox1*	COI_long22<br>Pat_R	ACWAAYCATAAAGATATTGGMAC<br>TCCAATGCACTAATCTGCCATATTA	1.55 kb	[3]<br>[4]
"	*nad1*<br>(+ 2nd half of *cob*)	steno_ND1_F1<br>steno_ND1_R1	GTAAAATGATTATGAGGRGGATT<br>CTTAGGGATAACAGCGTAATTYTTTYGG	2.0 kb	[3]
"	*cob* (1st half)	steno_cytB_F1<br>steno_cytB_R3	ATTTATATAGCAAGAGTAGCATCAAA<br>TTCTGGTTGAATATGAACTGGTGT	1.1 kb	[3]
"	*nad6*	steno_ND4_F2<br>steno_ND6_R1	CCTAAACCCAAAGCACCTTCACA<br>AATCCYCCTCATAATCATTTTAC	1.35 kb	[3]

Target organism	Target gene	probe name	Probe sequence (5'-3')	Labeling dye	Reference
Probes for fluorescence in situ *hybridization*					
Bacteria	16S rRNA	EUB338	GCTGCCTCCCGTAGGAGT	Cy3	[5]
EUB-Planctomycetes	16S rRNA	EUB338 II	GCAGCCACCCGTAGGTGT	Cy3	[5]
EUB-Verrucomicrobia	16S rRNA	EUB338 III	GCAGCCACCCGTAGGTGT	Cy3	[5]
-	16S rRNA	NON338	ACTCCTACGGGAGGCAGC	Cy3	[6]
Burkholderia-symbiont of Stenocephalidae	16S rRNA	StenoSymb440	ACTAATACTGTGGGGGGA	Cy3	[3]

[1] [1] Lane, D. J. 1991. In E. Stackenbrandt and M. Goodfellow (ed.), John Wiley and Sons, New York, NY. [2] 1. Spilker et al., 2009. J. Clin. Microbiol. 47: 2607–2610. [3] This study. [4] Simon et al., 1994. Ann. Entomol. Soc. Am. 87: 651–702. [5] Daims et al., 1999. Syst. Appl. Microbiol. 22: 434–444. [6] Manz et al., 1992. Syst. Appl. Microbiol. 15: 593–600.

Table S2. The number of *gyrB* gene clones in each OTU subgroups of the *Burkholderia* symbionts.

		Stenocephalidae-clade					SBE clade					PBE clade	
**Species**	**Insect ID**	**OTU1**	**OTU2**	**OTU3**	**OTU4**	**OTU5**	**OTU6**	**OTU7**	**OTU8**	**OTU9**	**OTU10**	**OTU11**	**Total**
Dicranocephalus agilis													
	Dag1	2	-	-	-	-	-	-	-	-	-	-	2
	Dag2	-	-	3	1	4	-	-	-	-	-	-	8
Dicranocephalus medius													
	Dme1	1	-	-	-	-	-	-	-	-	-	-	1
	Dme2	8	-	-	-	-	-	-	-	-	-	-	8
	Dme3	2	5	-	-	-	-	-	-	-	-	-	7
	Dme4	-	3	-	-	-	-	-	-	-	-	-	3
	Dme5	-	2	-	-	-	-	-	-	-	-	-	2
	Dme6	12	-	-	-	-	-	-	-	-	-	-	12
Dicranocephalus lateralis													
	Dla1	-	-	-	-	-	9	1	1	-	-	-	11
	Dla2	-	-	-	-	-	-	-	3	-	-	-	3
	Dla3	-	-	-	-	-	-	-	7	1	-	-	8
	Dla4	-	-	-	-	-	-	-	-	-	10	-	10
Dicranocephalus albipes													
	Dal1	-	-	-	-	-	-	-	-	-	-	13	13
	Dal2	-	3	-	-	-	-	-	-	-	-	-	3
	OTU total	25	13	3	1	4	9	1	11	1	10	13	**91**

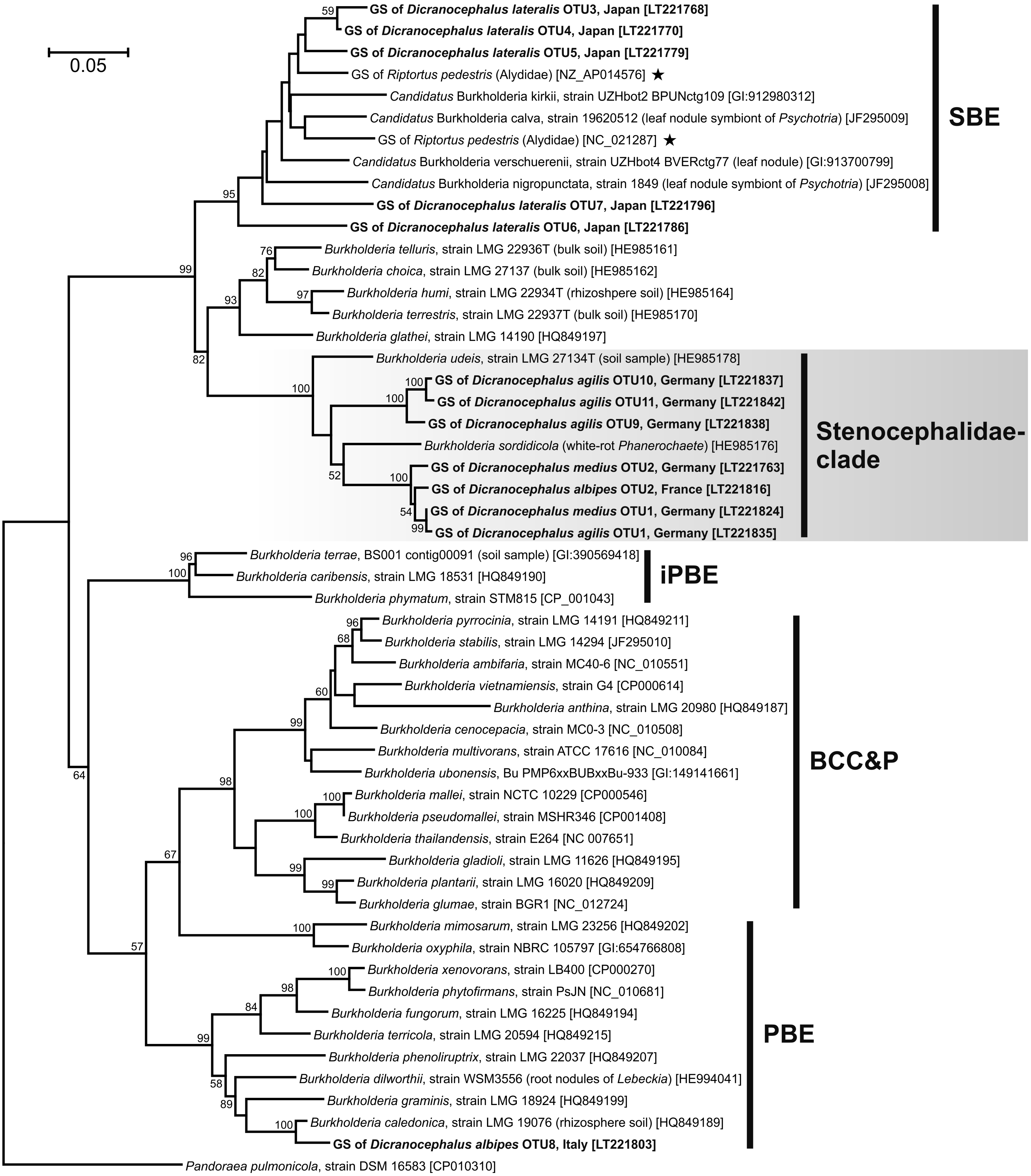
0.05
59
GS of Dicranocephalus lateralis OTU3, Japan [LT221768]
GS of Dicranocephalus lateralis OTU4, Japan [LT221770]
GS of Dicranocephalus lateralis OTU5, Japan [LT221779]
GS of Riptortus pedestris (Alydidae) [NZ_AP014576] ★
Candidatus Burkholderia kirkii, strain UZHbot2 BPUNctg109 [GI:912980312]
Candidatus Burkholderia calva, strain 19620512 (leaf nodule symbiont of Psychotria) [JF295009]
GS of Riptortus pedestris (Alydidae) [NC_021287] ★
Candidatus Burkholderia verschuerenii, strain UZHbot4 BVERctg77 (leaf nodule) [GI:913700799]
Candidatus Burkholderia nigropunctata, strain 1849 (leaf nodule symbiont of Psychotria) [JF295008]
95
GS of Dicranocephalus lateralis OTU7, Japan [LT221796]
GS of Dicranocephalus lateralis OTU6, Japan [LT221786]
SBE
76
Burkholderia telluris, strain LMG 22936T (bulk soil) [HE985161]
99
82
Burkholderia choica, strain LMG 27137 (bulk soil) [HE985162]
97
Burkholderia humi, strain LMG 22934T (rhizoshpere soil) [HE985164]
93
Burkholderia terrestris, strain LMG 22937T (bulk soil) [HE985170]
Burkholderia glathei, strain LMG 14190 [HQ849197]
82
Burkholderia udeis, strain LMG 27134T (soil sample) [HE985178]
100
GS of Dicranocephalus agilis OTU10, Germany [LT221837]
100
100
GS of Dicranocephalus agilis OTU11, Germany [LT221842]
GS of Dicranocephalus agilis OTU9, Germany [LT221838]
Burkholderia sordidicola (white-rot Phanerochaete) [HE985176]
100
GS of Dicranocephalus medius OTU2, Germany [LT221763]
52
GS of Dicranocephalus albipes OTU2, France [LT221816]
54
GS of Dicranocephalus medius OTU1, Germany [LT221824]
99
GS of Dicranocephalus agilis OTU1, Germany [LT221835]
Stenocephalidae-
clade
96
Burkholderia terrae, BS001 contig00091 (soil sample) [GI:390569418]
100
Burkholderia caribensis, strain LMG 18531 [HQ849190]
Burkholderia phymatum, strain STM815 [CP_001043]
iPBE
96
Burkholderia pyrrocinia, strain LMG 14191 [HQ849211]
68
Burkholderia stabilis, strain LMG 14294 [JF295010]
Burkholderia ambifaria, strain MC40-6 [NC_010551]
60
Burkholderia vietnamiensis, strain G4 [CP000614]
Burkholderia anthina, strain LMG 20980 [HQ849187]
99
Burkholderia cenocepacia, strain MC0-3 [NC_010508]
Burkholderia multivorans, strain ATCC 17616 [NC_010084]
64
Burkholderia ubonensis, Bu PMP6xxBUBxxBu-933 [GI:149141661]
BCC&P
98
100
Burkholderia mallei, strain NCTC 10229 [CP000546]
100
Burkholderia pseudomallei, strain MSHR346 [CP001408]
Burkholderia thailandensis, strain E264 [NC 007651]
67
Burkholderia gladioli, strain LMG 11626 [HQ849195]
99
99
Burkholderia plantarii, strain LMG 16020 [HQ849209]
Burkholderia glumae, strain BGR1 [NC_012724]
100
Burkholderia mimosarum, strain LMG 23256 [HQ849202]
57
Burkholderia oxyphila, strain NBRC 105797 [GI:654766808]
100
Burkholderia xenovorans, strain LB400 [CP000270]
98
Burkholderia phytofirmans, strain PsJN [NC_010681]
84
Burkholderia fungorum, strain LMG 16225 [HQ849194]
99
Burkholderia terricola, strain LMG 20594 [HQ849215]
PBE
Burkholderia phenoliruptrix, strain LMG 22037 [HQ849207]
58
Burkholderia dilworthii, strain WSM3556 (root nodules of Lebeckia) [HE994041]
89
Burkholderia graminis, strain LMG 18924 [HQ849199]
100
Burkholderia caledonica, strain LMG 19076 (rhizosphere soil) [HQ849189]
GS of Dicranocephalus albipes OTU8, Italy [LT221803]
Pandoraea pulmonicola, strain DSM 16583 [CP010310]

Fig. S1. Molecular phylogeny of the gut symbiotic bacteria of stenocephalid species based on the *gyrB* sequences. The tree displays a maximum likelihood (ML) phylogeny of eleven OTUs of the gut symbiotic bacteria (GS) identified from *Dicranocephalus albipes*, *D. agilis*, *D. lateralis* and *D. medius* together with selected representatives of the different *Burkholderia* groups. The alignment of 662 nucleotide sites of the *Burkholderia gyrB* gene was used. The gut symbionts of the stenocephalid species are shown in bold. The origins or sources of isolation of the *Burkholderia* strains/sequences are represented in parentheses. Accession numbers in the DNA database (DDBJ/ EMBL/GenBank) are shown in square brackets. *Burkholderia* gut symbionts of other stinkbug families are marked with an asterisk. The major *Burkholderia* clades (BCC&P, SBE and PBE) including the subclade “insect-associated PBE (iPBE)” and the "Stenocephalidae-clade" here described are indicated on the right. Bayesian (MrBayes) and neighbor-joining (NJ) analyses gave essentially the same results (data not shown). Bootstrap values higher than 50% are depicted at the nodes.
